# The gut microbiota-immune-brain axis in post-traumatic stress disorder: mechanistic integration and translational prospects

**DOI:** 10.3389/fimmu.2026.1859206

**Published:** 2026-06-12

**Authors:** Xianli Zheng, Dingpeng Li, Xiaoqiang Yao, Xixi Luo, Chunmei Gao, Xingke Yan

**Affiliations:** 1 Gansu University of Traditional Chinese Medicine, Lanzhou, Gansu, China; 2 The Affiliated Hospital of Gansu University of Traditional Chinese Medicine, Lanzhou, Gansu, China; 3 Gansu Provincial Second People’s Hospital, Lanzhou, Gansu, China; 4 The Affiliated Hospital of Northwest Minzu University, Lanzhou, Gansu, China

**Keywords:** brain axis, gut microbiota, immunity, microbiota-gut-brain axis, neuroinflammation, post-traumatic stress disorder, translational medicine

## Abstract

Post-traumatic stress disorder (PTSD) is a complex mental disorder triggered by severe traumatic events. Its pathophysiology involves not only abnormalities in fear memory circuits and neuroendocrine imbalances but also immune dysregulation and alterations in gut homeostasis. In recent years, the gut microbiota, as a crucial regulatory factor connecting the periphery and the central nervous system, has garnered widespread attention for its potential role in the development and progression of PTSD, offering a new integrative perspective for understanding this disorder. This article focuses on the “gut microbiota–immune–brain axis” framework, reviewing evidence related to changes in the composition and function of the gut microbiota in PTSD. It summarizes how these changes may influence neuroplasticity abnormalities and PTSD-related behavioral phenotypes through mechanisms involving microbial metabolite production, modulation of intestinal barrier integrity, immuno-inflammatory responses, regulation of neuroendocrine homeostasis, and blood-brain barrier dysfunction. However, these mechanistic pathways remain incompletely validated in human studies. Existing research suggests that this axis holds significant value in explaining the multisystem pathological features of PTSD. Nevertheless, challenges persist, including ambiguous causal relationships in microbiota–host interactions, limited direct clinical evidence, and insufficient translational research. Current evidence primarily stems from observational studies, preclinical models, and preliminary intervention studies. The explanatory power varies across these evidence levels: population studies primarily establish correlations, animal models facilitate mechanistic validation, metagenomic and metabolic analyses yield functional insights, while clinical intervention data remain exploratory. This article aims to elucidate the key molecular and systemic mechanisms underlying this axis in PTSD and to evaluate the potential translational value and practical limitations of microbial intervention and immune modulation strategies.

## 1 Introduction

Post-traumatic stress disorder (PTSD) is a mental disorder that may occur after exposure to severe traumatic events. Its core symptoms include traumatic re-experiencing, persistent avoidance, negative alterations in cognition and mood, and hyperarousal (1, 2). Previous studies have mostly explained the development of PTSD from the perspective of the central nervous system (CNS), focusing on functional abnormalities in fear memory-related brain regions such as the amygdala, hippocampus, and prefrontal cortex, as well as imbalances in the hypothalamic-pituitary-adrenal (HPA) axis (3, 4). However, with deepening research, PTSD is now considered not only limited to neural circuit abnormalities but may also be accompanied by widespread disturbances in multiple systems, including the immune, Endocrine, and autonomic nervous systems (5, 6). Among these, peripheral low-grade inflammation, immune imbalance, gastrointestinal symptoms, and alterations in gut microbiota composition have been reported in PTSD patients, suggesting that its pathophysiological process may involve a more complex brain-body interaction network (7).

In this context, the microbiota-gut-brain (MGB) axis has emerged as an important supplementary framework for understanding PTSD (8, 9). Existing studies suggest that gut microbiota dysbiosis may be linked to peripheral inflammatory responses and changes in central nervous function by affecting the production of microbial metabolites, intestinal barrier integrity, and host immune status (10, 11). In this process, the immune system is considered a key intermediary connecting gut microecological changes with brain functional abnormalities. Peripheral immune activation and the neuroinflammation it induces may further affect neuroplasticity, neurotransmitter signaling, and fear memory-related circuit functions, potentially correlating with the formation and maintenance of PTSD symptoms (12, 13). Therefore, the “gut microbiota-immune-brain axis” provides an insightful analytical framework for integrating the potential links among microbial changes, immune phenotype abnormalities, and Neuropsychiatric Symptoms in PTSD (14, 15). It should be noted that the evidence supporting this axis is still accumulating, and the strength of evidence at different levels is inconsistent. Overall, current studies suggest that PTSD may be accompanied by changes in gut microbiota composition, diversity, and functional aspects, and animal models and preliminary intervention studies have provided some support for this association (16). However, existing results are still influenced by factors such as sample size, study design, comorbid conditions, drug exposure, and individual lifestyle differences. The causal direction among microbial changes, immune abnormalities, and PTSD has not yet been fully clarified, and related translational evidence requires further validation (17, 18).

It is important to emphasize that gut microbiota dysbiosis, low-grade inflammation, and neuroinflammation are not unique to PTSD; similar changes are also observed in depression, anxiety, and chronic stress-related disorders. Therefore, this article does not simply regard these mechanisms as specific etiologies of PTSD but focuses on discussing how they intertwine with core features of PTSD, such as abnormal fear memory, threat processing imbalance, HPA axis abnormality, and autonomic hyperarousal, in the context of trauma exposure. Based on this, this article aims to review the possible links among gut microbiota changes, immune-inflammatory responses, and central nervous system functional abnormalities in PTSD within the framework of the “gut microbiota-immune-brain axis,” focusing on key aspects such as microbial metabolites, intestinal barrier function, peripheral immune activation, and neuroinflammation, and further analyze their potential value and practical limitations in the development of biomarkers and intervention strategies.

## 2 Evidence base for PTSD-related gut microbiota alterations

### 2.1 Human observational studies

Current human evidence on gut microbiota alterations in PTSD primarily comes from cross-sectional studies, systematic reviews, and a limited number of genetic epidemiological analyses. Overall, these studies suggest that individuals with PTSD may exhibit alterations in gut microbiota composition, diversity, and functional pathways, though consistency across studies remains limited (19). Existing systematic reviews have indicated that, compared to trauma-exposed controls, PTSD patients may show decreased α-diversity and reduced abundance of certain beneficial bacteria, such as Lachnospiraceae, which are associated with short-chain fatty acid production (20). Additionally, some individual studies have found alterations in the relative proportions of Bacteroidetes and Firmicutes, and changes in the abundance of genera such as Mitsuokella, Odoribacter, Catenibacterium, and Olsenella have been linked to the severity of PTSD symptoms. These findings suggest that PTSD-related microbiota alterations may not only reflect a binary “dysbiosis” status but may also involve more nuanced microbial characteristics associated with symptom heterogeneity. However, current human evidence primarily supports correlations and does not yet establish that these microbiota differences directly contribute to symptom formation (21).

Beyond taxonomic differences, human studies have begun to explore functional changes in the microbiome. Metagenomic analyses suggest that some individuals with PTSD may exhibit alterations in microbial functional pathways related to arginine utilization, citrulline, and ornithine metabolism (22). Theoretically, such changes could further impact host immune status, inflammatory response, and the production of neuroactive metabolites, thereby providing functional support for the link between “microbiota changes, metabolic abnormalities, and symptom manifestation” (23). Meanwhile, Mendelian randomization studies have proposed potential associations between specific bacterial genera and PTSD risk, offering preliminary clues about directional relationships, though these are insufficient to replace direct mechanistic evidence (24).

When interpreting human study results, the choice of control group is particularly critical. Compared to healthy controls, trauma-exposed individuals who did not develop PTSD provide greater explanatory power, as trauma exposure itself may influence dietary behavior, sleep rhythms, medication use, inflammatory status, and gut microbiota composition (25). Without trauma-exposed controls, observed microbiota differences are difficult to attribute specifically to PTSD pathology rather than the effects of trauma exposure itself (26). Overall, current human observational studies support that PTSD may be accompanied by certain gut microbiota alterations; however, the direction, stability, and clinical significance of these changes are modulated by factors such as sample size, study design, comorbidity status, and lifestyle differences, precluding their classification as a unified and stable phenotypic feature at this stage.

### 2.2 Animal model studies

Compared to human studies, animal models offer greater controllability for observing the dynamic relationship between traumatic stress and gut microbiota changes (27). Current research commonly employs single prolonged stress (SPS) or other stress paradigms to establish PTSD-like behavioral models, while simultaneously monitoring changes in microbiota composition, intestinal barrier function, and immune-inflammatory status (28, 29). Overall, these studies generally indicate that stress exposure can be accompanied by gut microbiota dysbiosis, occurring in parallel with abnormal fear memory, enhanced inflammation response, and other stress-related behavioral changes (30). For example, in a PTSD model using adolescent rats, stress led to changes in total gut microbiota abundance and the absolute abundance of specific phyla (19); in SPS models, alterations in microbiota composition were observed alongside intestinal barrier damage, low-grade inflammation, and behavioral abnormalities (31).

A key advantage of animal studies lies in their capacity to provide support for functional links between microbiota changes and PTSD-like phenotypes through intervention approaches (32). Existing research has shown that modulating gut microbiota or related immune pathways can, to some extent, improve PTSD-like behaviors, accompanied by changes in microbiota composition and metabolic status. For instance, interventions targeting the P2X7 receptor can alleviate behavioral abnormalities while affecting microbiota structure (33); probiotic treatment has been reported to improve microbiota dysbiosis, enhance intestinal barrier integrity, and reduce some stress-related behavioral abnormalities (34). Additionally, holistic microbiota remodeling strategies such as Fecal Microbiota Transplantation have been used for mechanistic validation, with some studies suggesting improvements in fear memory extinction deficits accompanied by changes in microbiota and neurochemical indicators (35). These results do not imply that “microbiota changes have been confirmed as a direct cause of PTSD,” but they at least suggest that in PTSD-like animal models, microbiota changes may not merely be a consequence of stress but could also participate in regulating related behavioral and immune phenotypes. However, this conclusion constitutes preclinical evidence and cannot be directly extrapolated to pathogenic mechanisms in human PTSD; therefore, caution is needed when interpreting animal model evidence. Different models primarily recapitulate specific behavioral or physiological domains of PTSD and cannot fully replicate the intrusive memories, complex cognitive appraisals, and social functional impairments characteristic of human PTSD (36, 37). Therefore, animal studies are better suited as important sources for mechanistic exploration and causal hypothesis generation, rather than as direct substitutes for human evidence.

### 2.3 Evidence strength, research contradictions, and methodological differences

Integrating current human observational studies and animal model research, it can be concluded that there is an association between PTSD and gut microbiota alterations, but the evidence base in this field still exhibits significant inconsistency. When interpreting this evidence, it is necessary to stratify it based on study design and explanatory power, rather than treating human observational studies, animal models, mechanistic inferences, and translational applications as evidence of equal strength. Overall, human longitudinal studies and intervention studies have relatively higher explanatory power, as they can more directly assess the temporal relationship between microbiota changes and symptom evolution. Human cross-sectional studies can provide clinical correlation clues but are limited in determining causal direction, while animal model studies aid in mechanistic validation and causal hypothesis generation. Furthermore, mechanistic inferences based on microbial metabolites, immune pathways, or studies of other psychiatric disorders primarily offer biological plausibility support, whereas microecological interventions and immunomodulatory strategies remain largely in the exploratory translational stage.

In human studies, some results support decreased α-diversity and reduced abundance of specific beneficial bacteria in PTSD patients, while others have found associations between certain bacterial genera and symptom severity (21). However, the specific microbiota differences, direction of changes, and effect sizes are not entirely consistent across studies. This indicates that while current human studies provide the most direct clinical evidence for the relationship between PTSD and gut microbiota alterations, the nature of this evidence remains primarily correlational rather than causal. In particular, most studies are cross-sectional, making it difficult to distinguish whether microbiota changes represent pre-trauma susceptibility traits, early post-trauma responses, or secondary alterations in chronic PTSD states (24).

Although animal studies more consistently show that stress exposure can be accompanied by microbiota changes and that microecological interventions can influence behavioral outcomes, the extent to which these results can be extrapolated to human PTSD still requires more direct evidence (38, 39). Compared to human studies, animal models offer advantages in controlling traumatic stress exposure, diet, genetic background, and environmental factors, allowing exploration of potential causal links between microbiota composition, intestinal barrier function, inflammatory response, and behavioral phenotypes. However, existing animal models primarily simulate certain behavioral or physiological features of PTSD, such as anxiety-like behavior, abnormal fear memory, or enhanced stress responses, and cannot fully replicate the intrusive memories, complex cognitive appraisals, long-term disease progression, and social functional impairments characteristic of human PTSD (40, 41). Therefore, animal studies are better suited as tools for hypothesis generation and preliminary validation rather than as direct substitutes for human evidence. Additionally, metagenomic and metabolic pathway studies have provided functional clues for understanding PTSD-related microbiota changes. For example, some studies suggest that individuals with PTSD may exhibit abnormalities in pathways related to amino acid metabolism, short-chain fatty acid production, or tryptophan metabolism, which helps transition from “microbiota composition differences” to an explanatory framework of “microbiota functional changes.” However, most such studies remain at the level of functional prediction or association analysis and require further confirmation through metabolomics, immunological assays, and intervention studies (42). In other words, metagenomic results can indicate potential mechanistic directions but cannot independently prove that these functional pathways directly participate in the onset and maintenance of PTSD.

The current inconsistency in evidence may primarily arise from several factors. First, study designs vary considerably. Human studies are mostly cross-sectional, making it difficult to determine whether microbiota changes represent pre-trauma susceptibility traits, early post-trauma responses, or secondary alterations in chronic PTSD states. Second, cohort heterogeneity and confounding factors are significant, including trauma type, disease stage, comorbidities such as depression or substance use, psychotropic medication exposure, dietary patterns, sleep, and lifestyle differences (43). Third, technical methods are not standardized, with substantial differences across studies in sample collection, storage, sequencing platforms, data processing, and bioinformatics analysis, directly affecting result comparability (44). Furthermore, the inclusion or exclusion of trauma-exposed control groups in human studies significantly influences the assessment of “PTSD-specific microbiota changes” (45). Compared to healthy controls, trauma-exposed individuals who did not develop PTSD offer greater utility in distinguishing between “effects of trauma exposure itself” and “PTSD-specific pathological changes,” thereby possessing higher methodological value in future research.

Therefore, a prudent conclusion is that current evidence supports the association of PTSD with specific alterations in gut microbiota composition and function; while this provides a foundation for discussing “microbiota-immune-brain” interactions, the data are insufficient to characterize these changes as a fully established, directionally consistent, and stable phenotype. In terms of evidence strength, the most reliable judgment at present is that “PTSD is associated with certain gut microbiota alterations”; a more cautious inference is that “microbiota changes may participate in PTSD pathological processes through metabolic, barrier, and immune-inflammatory pathways”; and “achieving clinical therapeutic translation through microecological or immune interventions” remains a promising but unverified direction. Prior to elaborating on related mechanisms, it is imperative to delineate the extent to which these changes are PTSD-specific rather than merely reflecting transdiagnostic biological alterations commonly observed in depression, anxiety, chronic stress, or other inflammation-related conditions.

### 2.4 PTSD specificity and transdiagnostic shared mechanisms: differentiation from other stress-related mental disorders

It should be noted that PTSD-related alterations in the gut microbiota-immune-brain axis are not entirely disease-specific. Phenomena such as low α-diversity, reduced abundance of short-chain fatty acid-producing bacteria, elevated IL-6 and TNF-α, changes in blood-brain barrier function, and microglial activation are also observed in depression, anxiety, chronic stress, inflammatory diseases, and some neurodegenerative disorders (46). Therefore, these changes are better understood as transdiagnostic features of stress-inflammation-brain dysfunction rather than as unique biomarkers of PTSD.

The relative specificity of PTSD may not reside in a single microbiota, cytokine, or neuroinflammatory indicator but rather in its specific pathological context and distinct combinatorial profiles. Compared to depression or generalized anxiety, PTSD is characterized by persistent threat processing abnormalities following explicit trauma exposure, impaired fear memory consolidation and extinction, trauma re-experiencing, hypervigilance, and avoidance behaviors (47). Therefore, for gut microbiota and immune-inflammatory changes to be considered more relevant to PTSD, they should be further demonstrated to have stable associations with post-trauma symptom trajectories, fear memory processing, amygdala-hippocampal-prefrontal circuit abnormalities, HPA axis feedback patterns, and autonomic hyperarousal states. From this perspective, the key to PTSD-related gut microbiota-immune-brain axis research is not to identify a single microbe or inflammatory marker “exclusive to PTSD” but to recognize characteristic combinations associated with symptom persistence, abnormal fear memory, and impaired stress recovery in the context of trauma exposure. For example, if certain microbiota functional changes are simultaneously linked to elevated peripheral inflammation, HPA axis feedback abnormalities, autonomic hyperarousal, and dysfunction in the amygdala-hippocampal-prefrontal circuit, they may have greater PTSD-related explanatory power than isolated α-diversity decreases or single cytokine elevations (48). Thus, PTSD-related gut microbiota-immune-brain axis alterations should be understood as relatively specific feature combinations within the context of trauma exposure, rather than single disease-specific indicators. The following discussion will further address relevant immune and neurobiological mechanisms while acknowledging their shared transdiagnostic nature (**Table 1**).

**Table 1:** Evidence base, strength of evidence, and interpretive boundaries for PTSD-related gut microbiome alterations.

Evidence type	Main findings	Evidence value	Main limitations	Interpretive boundaries	References
Population Observational Studies	PTSD patients may exhibit decreased α-diversity, reduction of some beneficial bacteria, and changes in the abundance of genera such as Mitsuokella, Odoribacter, Catenibacterium, and Olsenella.	Provides the most direct population-based correlative evidence linking PTSD to gut microbiome alterations.	Mostly cross-sectional studies with limited sample sizes and numerous confounding factors.	Primarily supports correlation; cannot yet indicate that microbiome differences directly contribute to symptom development.	
Metagenomic and Functional Pathway Studies	Some PTSD individuals may exhibit alterations in microbial functional pathways such as arginine utilization, and citrulline and ornithine metabolism.	Facilitates the transition from the “microbiome composition differences” framework to the “microbiome functional changes” explanatory framework.	Mostly involve functional prediction or correlational analyses, lacking metabolomic and immunological validation.	Can suggest potential mechanistic directions but cannot alone prove the involvement of these pathways in PTSD onset and maintenance.	(22, 23)
Genetic Epidemiological Studies	Mendelian randomization studies suggest potential directional associations between specific bacterial genera and PTSD risk.	Provides preliminary evidence regarding directional relationships between the microbiome and PTSD risk.	Cannot replace direct mechanistic studies; limited by instrumental variables and databases.	Can serve as a source for causal hypotheses but not as definitive causal evidence.	(24)
Animal Model Studies	In PTSD-like models such as SPS (single prolonged stress), stress exposure is associated with microbiome dysbiosis, intestinal barrier damage, enhanced inflammation, and behavioral abnormalities.	Advantageous for controlling traumatic stress, diet, and environmental factors, suitable for mechanistic exploration.	Animal models can only simulate some PTSD-like behaviors, making it difficult to replicate human intrusive memories and complex cognitive appraisals.	Constitutes preclinical evidence; cannot be directly equated with human PTSD pathogenic mechanisms.	(30, 31, 33)
Animal Intervention Studies	Probiotics, P2X7-related interventions, or FMT can improve some PTSD-like behaviors, accompanied by changes in microbiome and metabolic status.	Supports the possibility that microbiome changes may mediate behavioral and immune phenotypes.	Intervention effects mostly derived from animal experiments, with insufficient evidence for translation to human populations.	Suggests mechanistic plasticity but does not prove that related strategies already possess clinical efficacy for human PTSD.	(34, 35)
Control Group Design	Control groups comprising trauma-exposed individuals who did not develop PTSD help distinguish between trauma effects and PTSD-specific changes.	Enhances the interpretability of PTSD-related microbiome changes.	The number of related studies remains limited, with significant variations in cohort design.	Holds greater methodological value compared to healthy controls, but still requires longitudinal validation.	(25, 26)
Transdiagnostic Comparison	Low α-diversity, elevated IL-6/TNF-α, BBB alterations, and microglial activation are also observed in conditions like depression, anxiety, and chronic stress.	Helps avoid misattributing general stress or general inflammatory changes as PTSD-specific features.	Insufficient psychiatric disorder controls and trauma-exposed non-PTSD controls.	PTSD correlation is more likely reflected in a characteristic combination within the context of trauma exposure, rather than a single microbiome or inflammatory marker.	(46–48)
Comprehensive Judgment	PTSD may be accompanied by certain alterations in gut microbiome composition and function.	Provides an evidence base for subsequent discussions on the “microbiome-immune-brain axis”.	Inconsistent results, unclear causal direction, and non-uniform technical methodologies.	The most prudent current conclusion is “a certain correlation exists”; mechanistic roles and clinical translation require further verification.	(38–41)

## 3 Key pathways through which gut microbiota dysbiosis affects the immune system

### 3.1 Immunomodulatory effects of microbial metabolites

The gut microbiota regulates host immune homeostasis through various metabolites, including short-chain fatty acids (SCFAs), tryptophan metabolites, and indole derivatives, which have been extensively studied (49). These metabolites not only influence the local immune environment of the intestinal mucosa but also shape peripheral inflammatory states by regulating the intestinal epithelial barrier, innate immune cells, and adaptive immune responses (50). SCFAs such as butyrate, propionate, and acetate are primarily produced by the fermentation of dietary fiber by specific gut microbiota and play an important role in maintaining intestinal immune homeostasis (51). Among them, butyrate acts as a histone deacetylase (HDAC) inhibitor, promoting the differentiation and functional maintenance of regulatory T cells (Tregs) through epigenetic regulation, thereby exerting anti-inflammatory effects (52). Furthermore, SCFAs regulate the functions of macrophages and dendritic cells by activating G protein-coupled receptors such as GPR43 and GPR109A, contributing to the integrity of the intestinal epithelial barrier (53). Current research suggests that PTSD-related gut microbiota dysbiosis is often characterized by a reduced abundance of SCFAs-producing bacteria, thereby affecting SCFA production and their immunomodulatory effects (54). These results indicate that, in the context of PTSD-related dysbiosis, reduced SCFAs may weaken immune tolerance and promote the maintenance of low-grade inflammation; however, direct validation of this chain in PTSD patients remains limited, and some understanding is still primarily derived from general intestinal immunity research and related stress models.

In addition to SCFAs, tryptophan metabolism dysregulation is also considered an important pathway linking dysbiosis and immune abnormalities. Tryptophan metabolism primarily involves the kynurenine pathway, the 5-hydroxytryptamine pathway, and the indole pathway (55). Under inflammatory or stress conditions, the activity of indoleamine 2,3-dioxygenase (IDO) can be enhanced, driving more tryptophan towards the kynurenine pathway (56). This shift may increase the production of neuroactive metabolites such as quinolinic acid, which can act on N-methyl-D-aspartate (NMDA) receptors and is associated with microglial activation and the release of pro-inflammatory cytokines (57). Meanwhile, the reduced production of indole and its derivatives mediated by gut microbiota may weaken the activation of the aryl hydrocarbon receptor (AhR), thereby affecting intestinal epithelial barrier function, mucus secretion, and Treg induction (58). Therefore, based on stress models and general gut-brain axis research, PTSD-related microbiota changes may be associated with a shift in tryptophan metabolism towards pro-inflammatory and neuroactive directions, while also possibly being accompanied by reduced production of protective indole metabolites, thereby affecting intestinal immune homeostasis (59). However, direct evidence for the correspondence between tryptophan metabolic reprogramming and microbiota changes in PTSD patients is still insufficient, and related conclusions are more often based on research on inflammatory states, stress models, and other neuropsychiatric disorders (60).

Furthermore, there is biological coherence among microbiota metabolic imbalance, intestinal barrier damage, LPS translocation, and innate immune activation, which can serve as a candidate mechanistic chain to explain the source of peripheral inflammation in PTSD, but it has not yet been fully validated in human PTSD (61). In terms of evidence sources, the mechanisms by which SCFAs, tryptophan metabolites, and indole derivatives regulate immune homeostasis have been well supported in basic immunology and general gut-brain axis research (62); however, in the field of PTSD, population studies that directly link changes in microbiota composition, metabolite levels, peripheral immune activation, and symptom severity remain scarce (63). Therefore, these metabolic pathways are currently more suitable as candidate mechanisms for explaining PTSD-related immune metabolic abnormalities rather than as fully validated causal chains in human populations.

### 3.2 Changes in intestinal mucosal immunity and systemic immune phenotype

PTSD patients often exhibit peripheral immune imbalance and systemic low-grade inflammation, with elevated levels of various pro-inflammatory cytokines and acute-phase proteins in peripheral blood reported in some studies (64). In recent years, gut microbiota dysbiosis and changes in intestinal mucosal immunity are increasingly recognized as potential contributors to the development of this systemic immune phenotype, providing a new explanatory framework for understanding the source of peripheral inflammation in PTSD. At the local intestinal level, dysbiosis may first affect the integrity of the intestinal epithelial barrier and mucosal immune homeostasis (61). Animal studies suggest that stress exposure is associated with alterations in gut microbiota composition and may lead to downregulation of tight junction proteins such as occludin and ZO-1, thereby increasing intestinal permeability (65). In this context, bacterial metabolites and endotoxins can translocate more readily into the intestinal mucosal lamina propria, subsequently activating local innate immune cells. Existing research indicates that this process can be accompanied by increases in pro-inflammatory factors such as IL-1β, IL-6, and TNF-α, as well as a decrease in IL-10 and impaired Treg function, indicating weakened immune tolerance mechanisms (66, 67). Therefore, PTSD-related gut microbiota dysbiosis may participate in the formation of peripheral immune activation by altering the local inflammatory environment of the intestinal mucosa; however, this inference is currently more derived from stress animal models and general intestinal immunity research and cannot be simply regarded as a fully confirmed causal chain in PTSD patients.

The further extension of local intestinal mucosal inflammation to the systemic level is considered an important link connecting intestinal changes to abnormal systemic immune phenotypes (68). Theoretically, intestinal-derived inflammatory mediators and activated immune cells can enter the circulatory system, inducing immune cells such as peripheral blood monocytes to remain in a continuously activated state and promoting the release of more inflammatory factors (69). Consistent with this, some clinical studies have found elevated levels of IL-1β, IL-6, TNF-α, and C-reactive protein in the peripheral blood of PTSD patients, suggesting the presence of persistent low-grade inflammation (70). However, the sequential propagation of inflammation from the local intestinal mucosa to the peripheral blood and subsequently to the central nervous system remains largely hypothetical, relying on mechanistic deductions and indirect evidence; thus, the causal chain has not yet been fully elucidated. Additionally, some preclinical studies have suggested that modulating gut microbiota composition may improve intestinal mucosal inflammation and alleviate PTSD-like behaviors. For example, specific probiotic interventions can downregulate the expression of pro-inflammatory factors and, to some extent, improve behavioral abnormalities (71). These results indirectly support the potential significance of the “gut microbiota-intestinal mucosal immunity-systemic inflammation” pathway in PTSD-related pathology. Nevertheless, a more prudent judgment at this stage is that intestinal mucosal immune imbalance may be an important mediator linking microbiota changes to abnormal peripheral immune phenotypes, rather than a fully established unidirectional pathogenic starting point (72).

**Figure 1:**
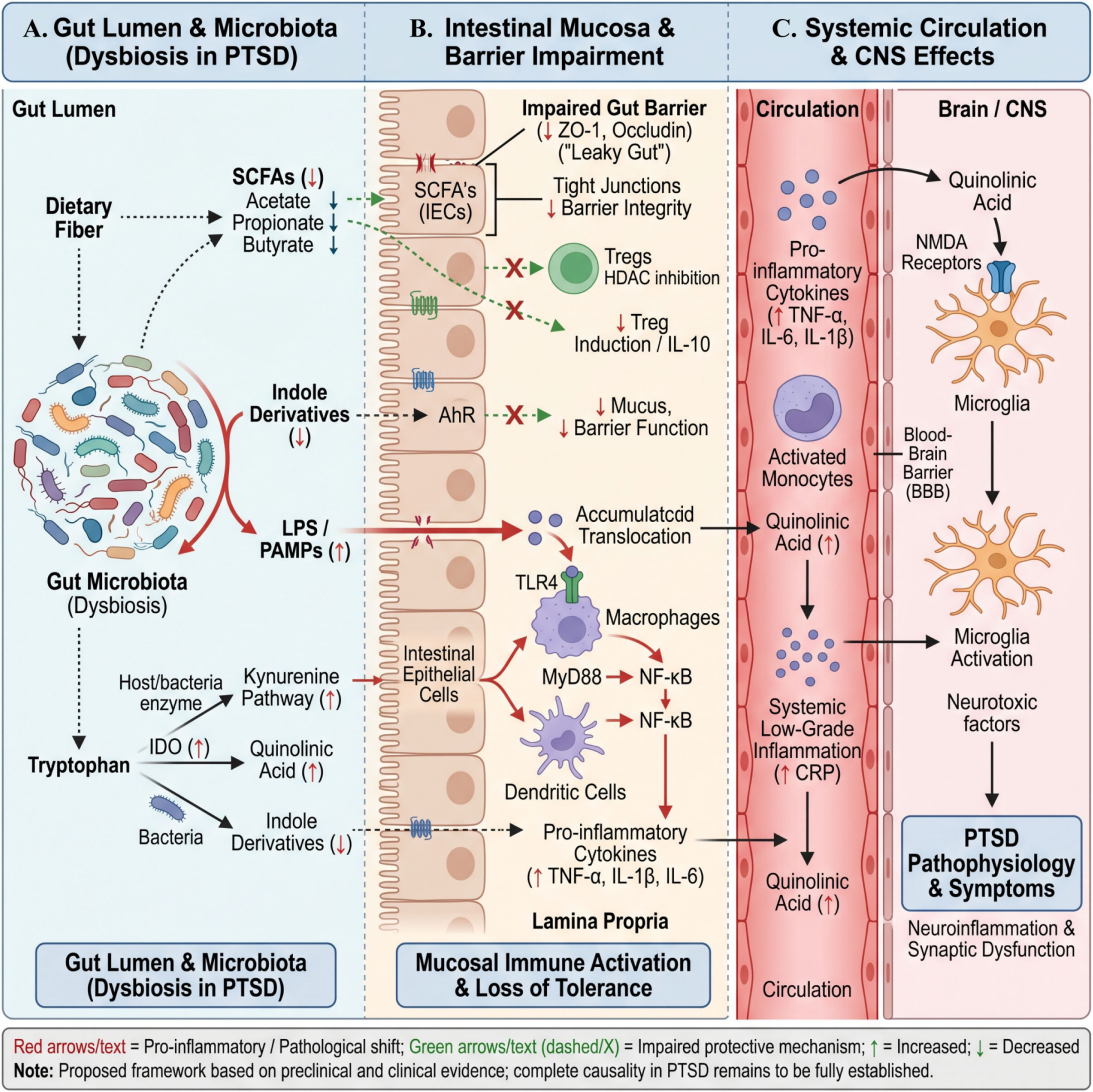
Potential links among gut microbiota dysbiosis, intestinal barrier damage, and systemic inflammation in PTSD. Microbiota imbalance may lead to reduced SCFAs and indole derivatives, increased LPS/PAMPs translocation, and the promotion of low-grade inflammation through activation of intestinal mucosal immunity. This mechanism provides a candidate framework linking intestinal changes to peripheral immune abnormalities, but its causal role in human PTSD still requires further validation.

Future research still requires longitudinal population studies, multi-omics analyses, and mechanistic experiments to further clarify its specific role in the development and progression of PTSD. In terms of evidence hierarchy, support for intestinal barrier damage and mucosal immune activation is relatively strong in animal models, but direct evidence in human PTSD remains insufficient. Although elevated peripheral inflammatory markers are common in population studies, the extent to which their origin is intestinal, they are driven by dysbiosis, and they directly impact PTSD symptoms still requires further validation through longitudinal cohorts, multi-omics integration, and mechanistic intervention studies (73). Specifically, intestinal barrier damage and mucosal immune activation have strong mechanistic support in stress animal models, but in human PTSD, there is still a lack of longitudinal studies simultaneously detecting intestinal barrier markers, microbial translocation markers, peripheral immune profiles, and clinical symptoms (74). Therefore, at this stage, it is difficult to determine the true contribution of intestinal-derived inflammation to peripheral inflammation in PTSD, nor can it be confirmed whether it is an upstream driving factor for disease onset. Notably, reduced SCFAs, tryptophan metabolic shift, LPS translocation, and peripheral low-grade inflammation are not exclusive to PTSD and can also be observed in depression, anxiety, and chronic stress states. Therefore, these mechanisms should more appropriately be understood as candidate pathways for post-traumatic stress-related immune metabolic imbalance. Their relevance to PTSD primarily lies in their potential interaction with abnormal fear memory, hypervigilance, and impaired stress recovery after trauma exposure, rather than acting as separate PTSD-specific mechanisms (**Figure 1**).

## 4 Transmission of immune signals to the brain and initiation of neuroinflammation

### 4.1 Effects of peripheral inflammatory factors on the blood-brain barrier and the brain

In the pathophysiology of PTSD, there may be an important link between peripheral immune activation and abnormalities in the central nervous system. Studies have suggested that PTSD patients may exhibit a persistent low-grade inflammatory state, with elevated levels of pro-inflammatory cytokines such as IL-6 and TNF-α in peripheral blood (75). These abnormal inflammatory signals need not strictly “enter the brain” through a single pathway; instead, they are more likely to influence the central environment through humoral, vascular, and neural pathways. For example, peripheral inflammatory factors can act on cerebral vascular endothelial cells and their receptors, inducing vascular-related inflammatory responses; some inflammatory signals may also affect the central nervous system through regions of reduced integrity of the blood-brain barrier, such as circumventricular organs (76). Therefore, from a mechanistic perspective, there is a biologically supported link between peripheral inflammation and brain dysfunction, but its specific intensity and temporal relationship in PTSD remain to be further clarified (77).

Changes in blood-brain barrier (BBB) function are considered an important pathway through which peripheral inflammation affects the central environment. Pro-inflammatory cytokines can act on cerebral vascular endothelial cells and pericytes, compromising tight junction integrity and barrier homeostasis, thereby increasing BBB permeability (78). In this context, inflammation mediators and some peripheral immune cells may more easily influence the brain parenchyma microenvironment and promote the initiation of central immune responses (79, 80). Thus, peripheral inflammation may affect brain function through pathways such as cerebral vascular endothelial responses, BBB functional changes, and central immune activation; however, in PTSD, this pathway is currently primarily a mechanistic hypothesis, lacking direct longitudinal evidence proving it drives core symptoms.

It should be distinguished that the mechanism by which peripheral inflammation affects BBB homeostasis is well-supported in basic neuroimmunology, but direct evidence in PTSD populations remains relatively limited. Currently, most studies support a correlation between peripheral inflammation and brain dysfunction, but are insufficient to prove that gut-derived inflammation directly drives central pathological changes in PTSD through BBB damage. Therefore, this pathway is better understood as a candidate mechanism linking peripheral immune imbalance and brain dysfunction, rather than a fully confirmed causal chain in population studies.

### 4.2 Effects of neuroinflammation on neuroplasticity and behavior

Neuroinflammation is hypothesized to contribute to PTSD-related abnormalities in neuroplasticity and further affect emotional, cognitive, and fear memory processing (81). Rather than repeating discussions on microglial activation and the release of inflammatory factors here, it is more productive to conceptualize neuroinflammation as an intermediate state affecting key brain circuit functions in PTSD. Currently, the most noteworthy brain regions are the hippocampus, amygdala, and prefrontal cortex, which are closely related to contextual memory, threat processing, and emotional regulation (82, 83).

In the hippocampus, neuroinflammation may affect contextual processing, memory integration, and fear memory extinction. PTSD patients often exhibit structural and functional abnormalities in the hippocampus, and the inflammatory microenvironment may weaken the hippocampus’s ability to encode contextual information and regulate traumatic memory by inhibiting neurogenesis, interfering with synaptic formation and repair, and reducing the expression of plasticity-related molecules such as brain-derived neurotrophic factor (BDNF) (84, 85). Therefore, hippocampal dysfunction may be associated with common deficits in contextual recognition, memory integration abnormalities, and insufficient fear extinction in PTSD.

In the amygdala, neuroinflammation may exacerbate threat processing and amplify fear responses by altering local neuronal excitability and synaptic transmission (86). The amygdala is a key brain region for threat recognition and fear memory processing. In the context of PTSD, neuroinflammation may be related to enhanced fear memory consolidation, generalization of emotional responses, and heightened sensitivity to threat cues (87); however, whether it directly leads to trauma re-experiencing, fear generalization, or extinction deficits in PTSD requires further validation through neuroimaging, inflammatory markers, and longitudinal symptom assessments (88–90). The prefrontal cortex is primarily involved in cognitive control and top-down regulation of the amygdala. If neuroinflammation affects the prefrontal cortex, it may impair its ability to regulate emotional responses and threat cues, leading to insufficient inhibition of trauma-related information, negative information bias, and emotional dysregulation (91, 92). Therefore, prefrontal dysfunction may be associated with persistent cognitive control deficits, emotional dysregulation, and avoidance behaviors in PTSD patients.

Overall, neuroinflammation does not act through a single molecule or a single brain region; instead, it is more likely to participate in the formation and maintenance of the PTSD symptom network by affecting the functional coordination among the hippocampus, amygdala, and prefrontal cortex. Studies from other neuropsychiatric disorders and inflammation models suggest that inflammatory states can broadly affect synaptic plasticity, neurotrophic support, and interregional connectivity, but the applicability of these findings to PTSD requires cautious interpretation in conjunction with direct research results (93). A more prudent judgment is that neuroinflammation may be an important mediator linking peripheral immune imbalance and brain dysfunction, and its relevance to PTSD is primarily reflected in its potential to affect circuits related to fear memory, threat processing, and emotional regulation, rather than serving as a standalone PTSD-specific mechanism (94).

**Figure 2:**
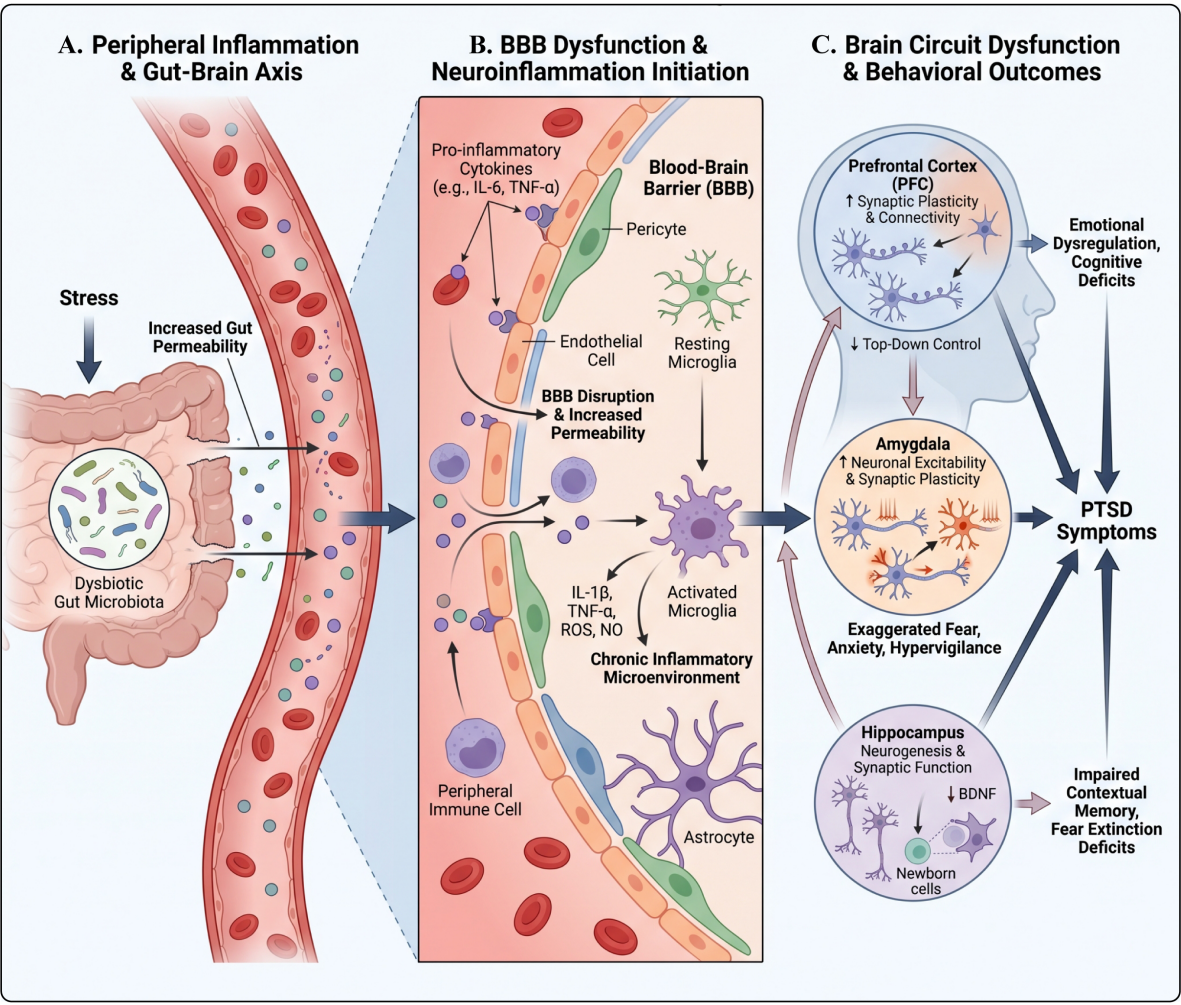
Potential links between peripheral inflammation, blood-brain barrier changes, and PTSD-related brain circuit abnormalities. Peripheral inflammatory signals may induce changes in the central immune environment by affecting the cerebral vascular endothelium and BBB homeostasis, thereby influencing brain regions involved in fear memory and threat processing, such as the hippocampus, amygdala, and prefrontal cortex. This framework offers a mechanistic explanation for the potential connection between peripheral immune imbalance and core PTSD symptoms, but its temporal relationship and causal role in human PTSD warrant further investigation.

From the perspective of evidence quality, basic research on neuroinflammation affecting synaptic plasticity and brain region connectivity is relatively robust, but its brain-region specificity and symptom correspondence in PTSD remain incompletely clarified. Current evidence cautiously suggests that while neuroinflammation modulates the functional status of circuits involving the hippocampus, amygdala, and prefrontal cortex, definitive links to specific symptoms such as trauma re-experiencing, fear generalization, or extinction deficits necessitate further validation via multimodal approaches (95). Additionally, blood-brain barrier changes, microglial activation, and neuroinflammation are also pathological processes shared by multiple neuropsychiatric disorders. For PTSD, the more noteworthy issue is not whether these changes are unique, but whether they preferentially affect circuits related to fear memory and threat processing, such as the hippocampus, amygdala, and prefrontal cortex, and whether they are associated with core PTSD symptoms like trauma re-experiencing, fear generalization, and extinction deficits (96, 97) (**Figure 2**).

## 5 HPA axis and the autonomic nervous system: key modulators of the gut microbiota-immune-brain axis

### 5.1 Bidirectional interaction of HPA axis function

The hypothalamic-pituitary-adrenal (HPA) axis is the core neuroendocrine system for coping with stress, and its functional abnormalities have long been considered a crucial component of the pathophysiology of PTSD (98). It should be noted that HPA axis abnormalities in PTSD exhibit significant heterogeneity, with different studies reporting variations in cortisol levels, glucocorticoid receptor sensitivity, and negative feedback regulation patterns (99). Therefore, rather than characterizing PTSD by a single fixed pattern of HPA axis imbalance, it is more appropriate to understand it as a differentiated remodeling of the stress regulatory network across individuals. This heterogeneity may also be related to trauma type, disease stage, comorbid conditions, and individual biological background.

Within the framework of the gut microbiota-immune-brain axis, the HPA axis is not merely a downstream component but may engage in a bidirectional regulatory interaction with the gut microbiota and its metabolites (100). Based on animal studies and general stress models, the gut microbiota may participate in regulating the body’s stress response by influencing glucocorticoid receptor sensitivity, local 11β-hydroxysteroid dehydrogenase activity, and gut homeostasis (101). Conversely, cortisol, the end product of the HPA axis, can in turn affect gut microbiota composition and the local immune environment, thereby forming a dynamic feedback loop (102). For example, some animal studies suggest that early-life adversity can be accompanied by transient changes in microbiota composition and subsequent HPA axis dysregulation, indicating that the gut microecology may be involved in the long-term shaping of stress response patterns (103). Additionally, microbial metabolites such as SCFAs may potentially modulate HPA axis activity indirectly by acting on the adrenal glands or central stress regulatory circuits (104, 105). However, direct evidence for these connections in PTSD patients remains limited, with most evidence currently derived from preclinical studies and mechanistic inferences.

Immune-inflammatory signals may also participate in the interaction between gut microbiota alterations and HPA axis dysfunction (106). Pro-inflammatory cytokines such as IL-1β and IL-6 can act on the hypothalamus and influence the release of corticotropin-releasing hormone (CRH), thereby altering HPA axis response patterns (107). The low-grade inflammatory state observed in PTSD may, to some extent, contribute to the maintenance of HPA axis abnormalities (108, 109); conversely, HPA axis dysregulation may also affect immune homeostasis and stress recovery capacity (110). Therefore, within this framework, the HPA axis is more suitably regarded as a key node regulating the microbiota, immune-inflammatory responses, and central stress response, rather than a unidirectional pathogenic pathway. Current evidence on microbiota influencing HPA axis feedback regulation primarily comes from animal studies and general stress models, and its specificity and causal significance in human PTSD require cautious interpretation. Future longitudinal population studies, simultaneously examining microbiota composition, metabolites, inflammatory markers, and HPA axis-related hormonal changes, are needed to further clarify their temporal relationships and true pathological weight.

### 5.2 Mediating role of the autonomic nervous system

The autonomic nervous system (ANS) serves as a crucial bridge connecting peripheral stress responses, gut environmental changes, and brain function regulation (111). In PTSD, ANS functional imbalance is typically characterized by increased sympathetic activity and decreased parasympathetic activity, particularly manifested as reduced vagal tone (112). This state of high arousal and low regulation is not only associated with symptoms such as hypervigilance but may also coexist with gut microbiota-immune-brain axis imbalance and play a modulating role. Nevertheless, it remains unclear whether the ANS serves as the primary mediator through which microbiota influence PTSD symptoms (113).

Sustained sympathetic excitation and decreased vagal tone may regulate the dynamic balance of the microbiota-immune-brain axis by affecting gut motility, barrier homeostasis, immune responses, and the cholinergic anti-inflammatory pathway (114–116). Current studies have suggested that PTSD patients often exhibit decreased vagally-mediated heart rate variability (vmHRV), a marker reflecting reduced parasympathetic regulatory capacity (117). However, it is prudent to regard vmHRV as a candidate indicator for assessing autonomic nervous function rather than a fully established, specific biomarker (118). Overall, PTSD patients often show reduced autonomic regulatory flexibility under resting and stress conditions, i.e., enhanced sympathetic response and insufficient parasympathetic recovery. This pattern may help explain their persistent hypervigilance and impaired post-stress recovery capacity (119).

The vagus nerve is also involved in the classic “cholinergic anti-inflammatory pathway.” When vagal efferent activity is enhanced, acetylcholine can act on peripheral immune cells and inhibit the release of pro-inflammatory cytokines, thereby limiting inflammatory amplification (120). Therefore, decreased vagal tone in PTSD may not only imply reduced emotional regulation and physiological recovery capacity but also impaired descending anti-inflammatory regulatory function. Rather than viewing ANS abnormalities as another independent inflammatory pathway, it is more appropriate to consider them as a neural modulation system that regulates the coupling between gut signals, peripheral immunity, and central stress responses.

**Figure 3:**
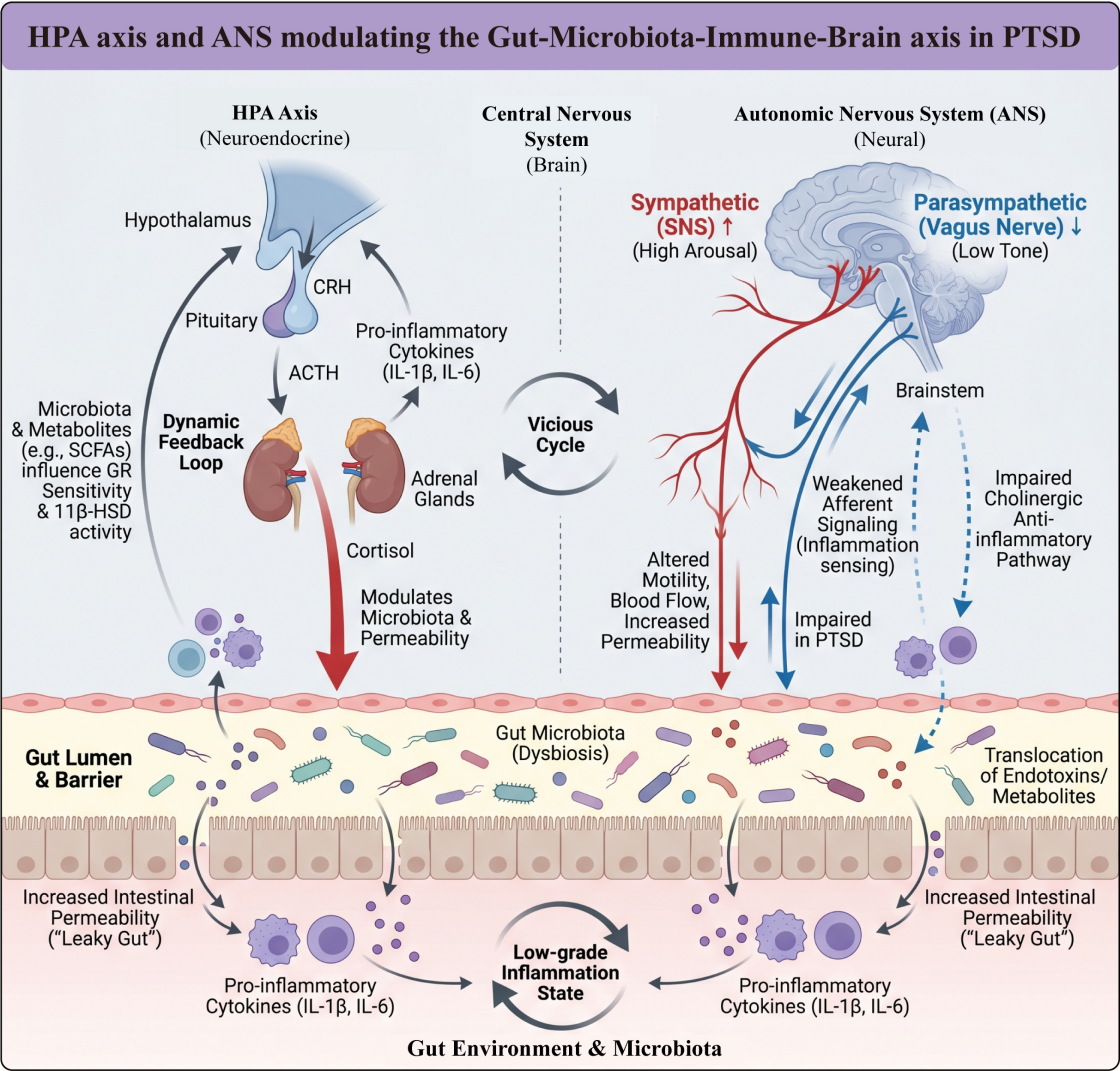
Modulatory effects of the HPA axis and autonomic nervous system on the gut microbiota-immune-brain axis in PTSD. The HPA axis and ANS can participate in regulating PTSD-related gut microbiota-immune-brain interactions by influencing stress hormones, gut motility, barrier homeostasis, immune regulation, and stress recovery capacity. This figure emphasizes their role as dynamic modulation systems, rather than viewing them as separate pathogenic pathways.

Overall, within the framework of this review, the autonomic nervous system is more suitably regarded as a neural modulation pathway between the gut microbiota, immune inflammation, and brain function. The HPA axis and ANS can influence the dynamic balance of the gut microbiota-immune-brain axis by regulating gut motility, barrier homeostasis, immune responses, and stress recovery capacity. The autonomic nervous system provides a neural pathway for bidirectional communication between gut signals and brain function. While current evidence in the PTSD field supports a correlation between autonomic dysfunction and symptom severity, it remains insufficient to establish that ANS changes are the primary mediator of microbiota dysbiosis affecting PTSD symptoms. Therefore, future longitudinal studies combining heart rate variability, inflammatory markers, microbiota data, and symptom trajectories are needed to clarify its regulatory role. It should be noted that HPA axis and autonomic nervous system abnormalities are not unique to PTSD, but PTSD may exhibit specific stress regulation patterns related to trauma exposure, persistent threat perception, and hypervigilance. Thus, within this framework, the significance of the HPA axis and autonomic nervous system lies not in providing single disease-specific markers, but in helping to explain the dynamic coupling between microbiota, immune inflammation, and trauma-related brain circuit abnormalities (**Figure 3**).

## 6 Clinical biomarker potential: from microbiome to immune fingerprint

### 6.1 Microbiome as an auxiliary tool for diagnosis and stratification

Alterations in the gut microbiome in PTSD provide new research directions for identifying objective biological stratification indicators. Current studies suggest that PTSD patients may exhibit decreased microbial diversity, altered abundance of specific genera, and imbalances in microbial metabolic functions (121). Therefore, the microbiome is more appropriately regarded as a potential stratification marker in the study of PTSD biological heterogeneity, rather than a mature tool ready for clinical diagnosis. For example, systematic reviews indicate that compared to trauma-exposed controls, PTSD patients may show reduced α-diversity and decreased abundance of families such as Lachnospiraceae (74); other studies suggest that the relative composition of Bacteroidetes and Firmicutes, as well as changes in the abundance of genera such as Mitsuokella, Odoribacter, Catenibacterium, and Olsenella, may correlate with PTSD symptom severity (45). Beyond compositional changes, metagenomic studies also suggest that some PTSD individuals may exhibit alterations in microbial functional pathways related to arginine utilization, citrulline and ornithine metabolism, offering insights into metabolic and immune differences across PTSD subtypes (122, 123). However, a more cautious assessment at this stage is that microbiome features theoretically hold auxiliary stratification value but are not yet sufficient to form stable, universally applicable clinical diagnostic tools. In PTSD microbiome research, the inclusion of trauma-exposed controls is particularly important. Since trauma exposure itself can affect gut microbiota, eating behavior, sleep patterns, and inflammatory status, comparing PTSD patients only with healthy individuals hinders the differentiation between “trauma exposure effects” and “PTSD-specific changes” (124).

Overall, PTSD-related microbiome biomarkers remain in the exploratory phase. No single genus, α-diversity index, or microbial functional pathway should be prematurely regarded as a diagnostic biomarker for PTSD, nor can any of these alone specifically differentiate PTSD from depression, anxiety, or other chronic stress-related disorders. Future research should focus more on the combinatorial relationships between microbial functional changes and symptom dimensions, immune-inflammatory profiles, HPA axis function, and autonomic nervous status in the context of trauma exposure.

### 6.2 Clinical application of peripheral immune-inflammatory profiles

Similar to microbiome research, peripheral immune-inflammatory profiles are considered an important entry point for understanding PTSD biological heterogeneity. Current studies generally suggest that PTSD is often accompanied by a state of chronic low-grade inflammation, with elevated levels of various pro-inflammatory cytokines and acute-phase reactants in peripheral blood (125). Among these, IL-6, TNF-α, and C-reactive protein are frequently reported indicators, while changes in anti-inflammatory effectors such as IL-10 show some variability across studies (126). Therefore, a more prudent statement is that PTSD may manifest as a pro-inflammatory peripheral immune state rather than a fixed pattern of “high IL-6, low IL-10.” In addition to soluble inflammatory factors, enhanced reactivity of monocyte pattern recognition receptors, such as Toll-like receptor 4 (TLR4), may also reflect increased sensitivity to inflammatory stimuli (127–129). It is important to emphasize that the evidence for peripheral immune-inflammatory profiles is slightly stronger than that for microbiome biomarkers, as some inflammatory factors and acute-phase reactants have been more frequently reported in PTSD populations. However, these indicators lack disease specificity and are susceptible to influences from infection, obesity, sleep disorders, psychotropic medications, metabolic status, and comorbid conditions. Therefore, peripheral immune indicators are more suitable as components of candidate biological phenotypes and combined stratification models rather than being used alone for PTSD diagnosis, prediction, or monitoring.

**Figure 4:**
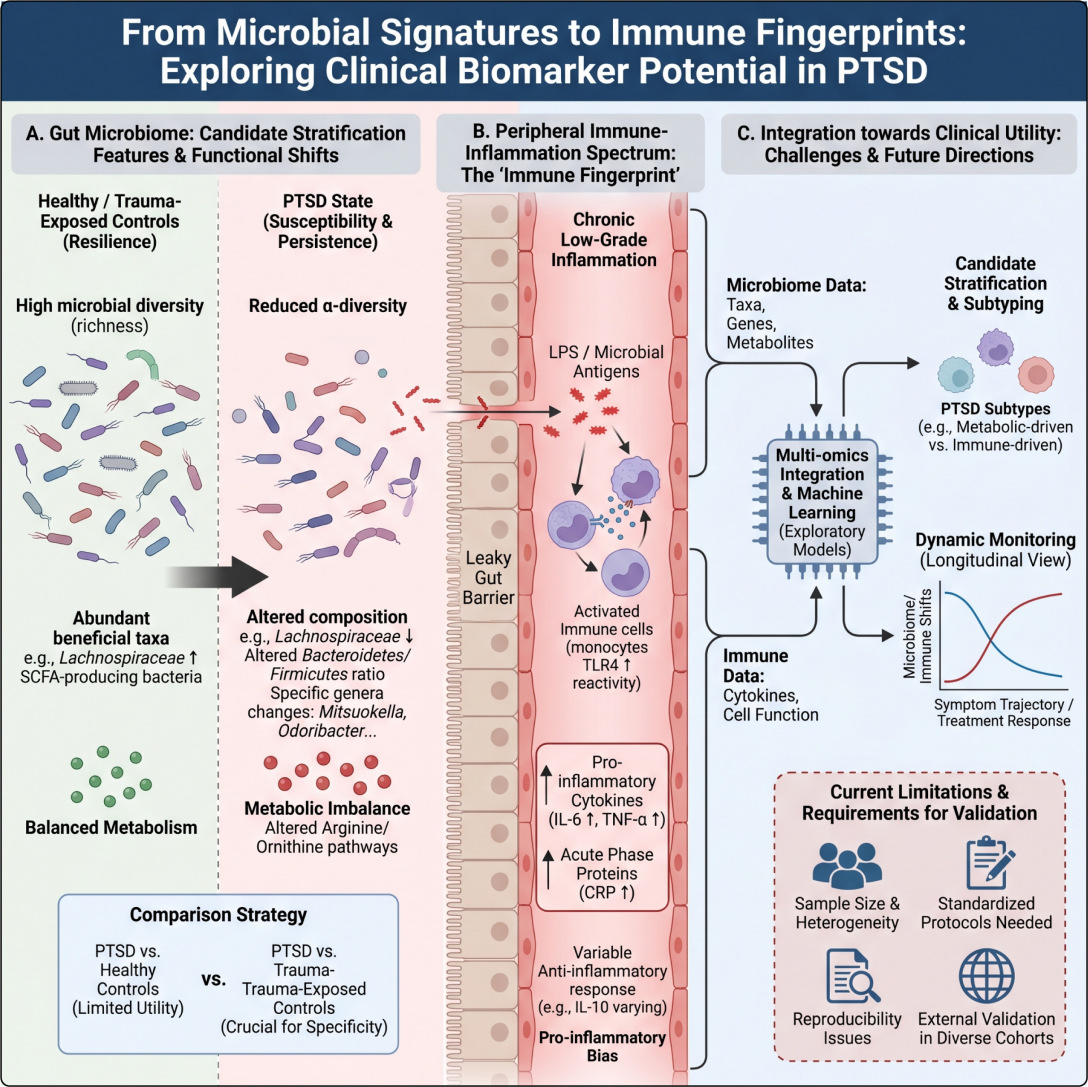
From microbial signatures to immune fingerprint: a potential integrative framework for PTSD clinical biomarkers. PTSD-associated microbiome and peripheral immune-inflammatory indicators can provide candidate clues for patient stratification, disease course monitoring, and treatment response prediction. Single microbial or inflammatory indicators lack diagnostic specificity; future efforts should integrate trauma exposure, symptom dimensions, microbial function, immune profiles, HPA axis, and autonomic nervous indicators to build multi-dimensional models. This direction still requires large-sample longitudinal cohorts, standardized detection protocols, and external validation support.

To enhance stratification capability, recent studies have attempted to integrate microbiome and immune-inflammatory profiles (15). Theoretically, combining microbial diversity, abundance of specific genera, cytokine levels, CRP, and microbial functional pathways may help generate multi-dimensional phenotypic stratification hypotheses, but these still require validation in independent cohorts and longitudinal predictive validity (130–132). However, such models are currently in the exploratory phase, limited by sample size, cohort heterogeneity, variable selection, and insufficient external validation, and remain far from being generalizable Clinical translation tools (133). From a disease-specific perspective, there is currently insufficient evidence to suggest that any single cytokine, acute-phase reactant, barrier function indicator, or immune cell characteristic can specifically differentiate PTSD from depression, anxiety, or other chronic stress-related disorders (134). Future biomarker studies should establish multi-dimensional stratification models incorporating trauma exposure information, symptom dimensions, microbial function, immune-inflammatory profiles, HPA axis, and autonomic nervous indicators, while including psychiatric disease controls and trauma-exposed non-ill controls, to identify biological features associated with PTSD persistence rather than general stress responses (135). Thus, the primary value of current microbiome and immune-inflammatory profiles lies not in providing single diagnostic biomarkers but in helping construct testable stratification hypotheses. Only when these indicators are replicated in independent cohorts and can predict symptom trajectories, treatment responses, or post-trauma recovery outcomes can they potentially advance into the Clinical transformation stage (**Figure 4**).

## 7 Intervention strategies based on the gut microbiota-immune-brain axis

**Figure 5:**
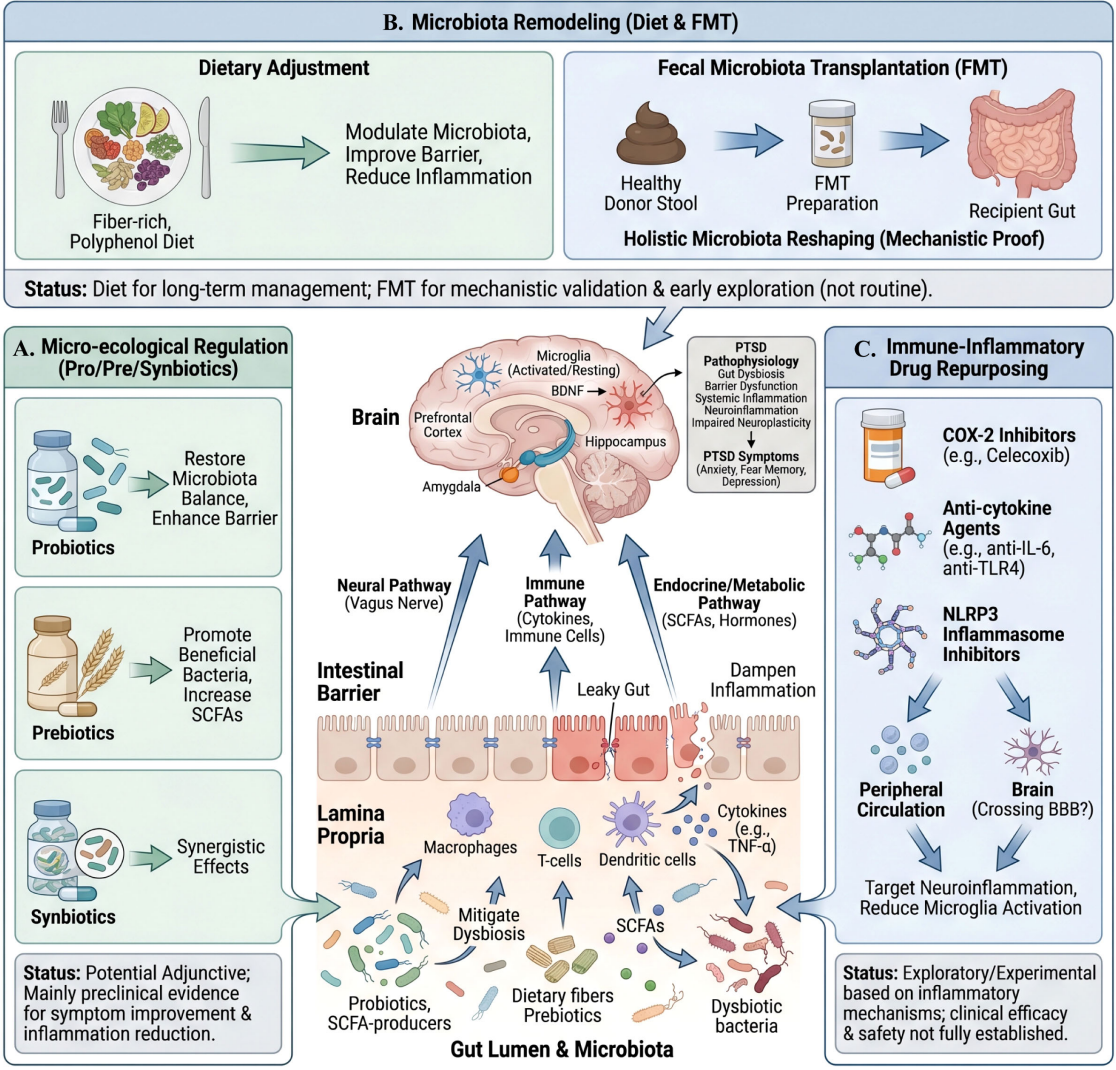
Potential intervention strategies for PTSD targeting the gut microbiota-immune-brain axis. Interventions centered on the gut microbiota-immune-brain axis mainly include three categories: microbial supplementation, microbiota remodeling, and immunomodulation/drug repurposing. Probiotics, prebiotics, dietary adjustments, FMT, and anti-inflammatory drugs may affect PTSD-related phenotypes by modulating microbiota composition, metabolites, intestinal barrier function, and inflammatory responses. These strategies suggest that this axis has a certain degree of plasticity, but most of the current evidence is still based on preclinical studies, mechanistic inferences, or exploratory clinical studies, and should not be conflated with clear clinical efficacy evidence for human PTSD.

Based on the aforementioned evidence, the gut microbiota-immune-brain axis provides a new entry point for understanding the multisystem pathology of PTSD and exploring intervention strategies. However, when discussing relevant strategies, a distinction should be made between “mechanistic intervenability” and “clinical applicability.” Currently, probiotics, prebiotics, Fecal Microbiota Transplantation (FMT), and anti-inflammatory interventions have demonstrated potential efficacy mainly in animal models or small-sample studies, suggesting that this axis has a certain degree of plasticity, but it is not yet sufficient to support it as a mature clinical treatment plan. Related research still faces limitations such as strain selection, doses, treatment course, patient stratification, safety, and insufficient long-term follow-up. Therefore, the interventions described in this section are more suitable as candidate research directions for potential adjunctive therapies and tools for mechanism validation, rather than alternatives to standard psychotherapy or first-line pharmacotherapy. At present, relevant strategies can be broadly categorized into three groups: microbial supplementation, microbiota remodeling, and immunomodulation combined with drug repurposing (**Figure 5**).

### 7.1 Probiotic, prebiotic, and synbiotic interventions

Microecological interventions centered on the gut microbiota-immune-brain axis are among the most prominent areas of focus in current PTSD intervention research. Among them, probiotics, prebiotics, and synbiotics are considered likely to have some impact on PTSD-related symptoms by modulating microbiota composition, improving intestinal barrier function, and alleviating low-grade inflammation (136). The following provides an overview from three aspects: their mechanisms of action, existing research basis, and application limitations.

In animal models, there is relatively more evidence for probiotic interventions. Studies have shown that in PTSD-like models such as single prolonged stress (SPS), specific probiotic treatments can restore microbiota homeostasis, enhance intestinal barrier integrity, and reduce anxiety-like and depression-like behaviors (29). Some studies have also observed that probiotic intervention is accompanied by the restoration of BDNF levels, downregulation of pro-inflammatory cytokines, and improvement in intestinal ultrastructure (137). These results suggest that probiotics may affect PTSD-related behavioral phenotypes by modulating the interaction between microbiota, immunity, and neuroplasticity. However, their main value lies in suggesting that “microbiota modulation may affect PTSD-like phenotypes,” rather than directly proving that probiotics have a clear therapeutic effect on human PTSD.

Similar to probiotics, prebiotics primarily indirectly affect inflammation and brain function by promoting the growth of beneficial bacteria and the production of their metabolites. An important mechanism is enhancing the production of short-chain fatty acids (SCFAs), thereby improving intestinal barrier homeostasis and regulating immune responses (138). In human research, a limited number of pilot studies have explored the use of prebiotics as an adjunct to standard treatments, such as Cognitive behavioral therapy, suggesting that certain individuals with PTSD may benefit from such interventions (139). However, the effect size, population differences, and specific mechanisms of action have not yet been consistently confirmed.

Synbiotics combine the theoretical advantages of probiotics and prebiotics, offering the potential for synergistic regulation by simultaneously supplementing beneficial bacteria and providing metabolic substrates. Existing preclinical reviews suggest that synbiotics may have some potential for microbiota remodeling and behavioral improvement, but the number of related studies is still small, and most lack systematic validation of PTSD-specific mechanisms (140). Overall, probiotics, prebiotics, and synbiotics can be explored as candidate research directions for adjunctive PTSD interventions, but their practical application is still influenced by factors such as strain selection, doses, treatment course, comorbidities, dietary background, and long-term safety. Therefore, microecological supplementation strategies are currently more appropriately understood as adjunctive intervention hypotheses with mechanistic plasticity, rather than clinically established treatment plans.

### 7.2 Dietary adjustments and fecal microbiota transplantation

Dietary adjustments and FMT represent two different approaches to microbiota intervention: the former is a relatively mild, long-term gradual adjustment, while the latter is a more direct holistic microbiota remodeling. They respectively reflect the different values of long-term microecological management and mechanistic microbiota reconstruction in PTSD intervention exploration.

Dietary intervention is a relatively low-risk, long-term microecological modulation method that has received widespread attention in research on the gut microbiota-immune-brain axis. Studies have suggested that dietary patterns rich in fiber and polyphenols may help increase the abundance of short-chain fatty acid-producing bacteria, improve intestinal barrier function, and alleviate peripheral low-grade inflammation (141). Based on this mechanism, dietary adjustments are postulated to exert a regulatory effect on PTSD-related microbiota imbalance and inflammatory states. However, direct evidence for specific dietary patterns, especially the Mediterranean diet, in the PTSD population is still limited, and existing inferences are largely based on general microecological and neuropsychiatric disease research (142). Therefore, dietary adjustments are better positioned as a promising avenue for long-term health management and adjunctive intervention, rather than as a definitive disease-specific treatment protocol.

Compared with dietary adjustments, FMT represents a more direct method of microbiota remodeling. In PTSD-related preclinical studies, FMT has been used to explore the potential link between microbiota changes and behavioral phenotypes (143). Some animal studies have shown that in PTSD-like models such as SPS, FMT may improve fear memory extinction deficits and some anxiety-like behaviors, accompanied by changes in microbiota composition and certain neurochemical indicators (28). These results support the research value of FMT as a mechanism validation tool, i.e., observing the linked changes in behavior, inflammation, and neuroendocrine function through holistic remodeling of the gut microbiota. However, the clinical translation of FMT in PTSD still requires caution. Its application involves not only donor screening, preparation processes, and administration methods but also issues such as safety, ethical regulation, long-term follow-up, and applicable populations (144, 145). Overall, both dietary adjustments and FMT suggest that the gut microbiota is modifiable, but their positioning differs: dietary adjustments are better suited for long-term adjunctive management, while FMT is currently more suitable as a tool for mechanistic research and proof-of-concept studies, rather than a proven effective PTSD treatment strategy.

### 7.3 Drug repurposing targeting immune-inflammatory pathways

In addition to directly modulating the gut microbiota, drug repurposing targeting immune-inflammatory pathways is also a research direction worth attention. Given that PTSD is often accompanied by peripheral low-grade inflammation and neuroinflammation-related changes, existing anti-inflammatory drugs or immunomodulatory drugs have been proposed as potential therapeutic interventions (146). Unlike microecological interventions, the advantage of drug repurposing lies in the existing safety and pharmacological basis of some candidate drugs, but their indications, target populations, and real additional benefits in PTSD still need further clarification.

Some studies have attempted to evaluate the potential role of traditional anti-inflammatory drugs in PTSD. For example, the selective Cyclooxygenase-2 (COX-2) inhibitor Celecoxib has attracted attention due to its anti-inflammatory effects, and small-sample studies suggest it may produce some improvement in certain PTSD symptoms (147). However, these results are still preliminary and insufficient to confirm the consistent efficacy of anti-inflammatory therapy in PTSD, nor can they alone establish that neuroinflammation constitutes a clear reversible therapeutic target (148). A more reasonable understanding is that these studies provide some support for “inflammation participating in the pathological process of PTSD,” but their clinical significance still needs validation through larger samples and clearer stratification of inflammatory phenotypes (149). Therefore, traditional anti-inflammatory drugs are currently more suitable as candidates for exploratory research in drug repurposing, rather than interventions that can already be recommended for routine PTSD treatment.

Strategies targeting more specific inflammatory pathways, such as interventions targeting IL-6, TLR4, or the NLRP3 inflammasome, are currently mainly at the stage of mechanistic inference or preclinical research (150). Theoretically, these targets are associated with stress-related inflammatory amplification, microglial activation, and neuroimmune imbalance, thus having some research appeal; however, direct evidence in the PTSD population is still weak, and the blood-brain barrier permeability, long-term safety, and applicable subgroups of related drugs are also unclear (151, 152). In recent years, multi-omics and network medicine methods have also been used to predict potentially repurposable drugs. These methods help screen candidate intervention targets from disease-related molecular networks, but currently mainly have the significance of proposing new hypotheses for repurposable intervention targets (153).

Overall, the value of drug repurposing in PTSD mainly lies in providing exploratory supplementary ideas for inflammation- and neuroimmune-oriented interventions, rather than replacing standard psychotherapy and first-line pharmacotherapy. Future research should prioritize identifying which PTSD subgroups have reproducible inflammatory phenotypes and, based on this, evaluate whether immunomodulatory interventions have real additional benefits. Therefore, immunomodulatory strategies should currently be regarded as potential adjunctive approaches based on mechanistic rationale, rather than PTSD treatment plans fully supported by clinical efficacy evidence (**Table 2**).

**Table 2:** PTSD intervention strategies based on the gut microbiota–immune–brain axis: main pathways, evidence levels, and practical limitations.

Intervention category	Representative strategy	Primary mechanism of action	Current evidence level	Research significance or potential value	Main limitations	Reference
Microecological supplementation strategy	Probiotics	Regulate gut microbiota composition, improve intestinal barrier integrity, downregulate pro-inflammatory factors, and may affect BDNF and neuroplasticity.	Predominantly animal studies with a few early human studies.	May serve as a candidate research direction for PTSD adjunctive intervention.	Strain, dosage, treatment duration, and target population are not yet standardized; human evidence is limited.	(136, 137)
	Prebiotics	Promote the proliferation of beneficial bacteria and the production of their metabolites, enhance SCFA production, and improve intestinal barrier and immune homeostasis.	A small number of pilot human studies plus preclinical research.	May serve as a research hypothesis for standard treatment adjunctive strategies.	Efficacy magnitude, mechanistic chain, and population differences remain unclear.	(138, 139)
	Synbiotics	Combine probiotics and prebiotics to support both bacterial supplementation and substrate provision.	Primarily preclinical reviews and early exploration	Theoretically possible synergistic regulation.	PTSD-specific research is scarce; mechanism and efficacy evidence are insufficient.	(140)
Microbiota remodeling strategy	Dietary modification	Increase the abundance of short-chain fatty acid-producing bacteria, improve intestinal barrier function, and reduce low-grade inflammation.	Mostly derived from general microbiota and neuropsychiatric disease research; direct PTSD evidence is limited.	Low risk, can serve as a research direction for long-term adjunctive management	There is insufficient specific clinical evidence for PTSD, and compliance and individual differences have a significant impact.	(141, 142)
	FMT	Holistically remodel the gut microbiota, verifying the link between microbiota changes and fear memory, inflammation, and neuroendocrine activity.	Primarily animal studies and mechanistic validation.	Valuable for elucidating the potential causal relationship between microbiota and behavior.	Safety, donor screening, preparation procedures, long-term effects, and ethical oversight remain to be clarified.	(143–145)
Immuno- modulation and drug repurposing	Traditional anti-inflammatory drugs (e.g., COX-2 inhibitors)	Inhibit peripheral inflammation and certain neuroinflammatory pathways	Small-sample clinical studies plus mechanistic reasoning.	Provides intervention clues for the involvement of inflammation in PTSD.	Efficacy is variable; insufficient to support routine application.	(147–149)
	Targeting IL-6, TLR4, NLRP3, and other pathways.	Target stress-related inflammatory amplification, microglial activation, and neuroimmune imbalance.	Primarily preclinical research or mechanistic reasoning.	May serve as a candidate mechanism direction for future precision intervention.	Direct human evidence is insufficient; blood-brain barrier permeability, safety, and applicable subgroups are unclear.	(151, 152)
	Multi-omics/network medicine- driven drug repurposing.	Screen candidate drugs and targets from disease-related molecular networks.	Hypothesis generation stage	Helps propose new repurposable intervention target hypotheses.	Still requires mechanistic experiments and clinical trial validation.	(153)

## 8 Evidence gaps, methodological challenges, and translational pathways

### 8.1 Limitations and challenges of current research

Overall, the main limitation of current research is not the lack of potential mechanisms, but the lack of a high-quality evidence chain that can sequentially link “microbiome alterations—immune abnormalities—brain function changes—PTSD symptoms”. Existing literature often reports separately on gut microbiota composition differences, elevated inflammatory markers, or neural circuit abnormalities, but rarely validates the temporal relationships and causal connections among these levels synchronously within the same PTSD cohort. Therefore, future research needs to shift from single-level descriptions to cross-level mechanism validation. Another issue to note is that some mechanistic conclusions are often pieced together from different evidence sources. For example, changes in gut microbiota, elevated inflammatory markers, altered BBB function, and brain circuit abnormalities may come from different research systems and cannot be simply considered as having been continuously validated in the same PTSD population (154). Hence, future reviews and original studies should more precisely distinguish between direct evidence, indirect evidence, and evidence from mechanistic extrapolation.

The core issue in this field is not whether associations exist between the gut microbiota, immune system, and PTSD, but whether the strength, causal direction, and clinical translatability of these associations are sufficiently clear. Existing evidence exhibits distinct hierarchical differences: population-based observational studies primarily provide clinical correlations, animal models support mechanistic feasibility, metagenomic and metabolomic studies offer functional clues, and interventional studies are used to test the plasticity and translational potential of this axis. The questions answered by different evidence levels are not the same, so mechanistic findings in animal models, cross-sectional population associations, and clinical intervention prospects should not be interpreted as evidence of equal strength. Overall, current research faces the following five types of challenges. First, the causal direction remains unclear. Most current clinical studies are cross-sectional in design, only revealing correlations between gut microbiota dysbiosis, immune abnormalities, and PTSD status, making it difficult to distinguish whether these changes are pre-traumatic susceptibility factors, disease triggers, or secondary manifestations during the disease course (42, 155). Thus, moving from “correlation” to “causation” remains a paramount methodological challenge in this field. Second, there is significant clinical heterogeneity and numerous confounding factors. PTSD patients vary considerably in trauma type, illness stage, symptom profile, comorbidities, and medication exposure (156), while factors such as diet, lifestyle, sex, genetic background, metabolic status, and lifetime trauma burden also influence gut microbiota composition and inflammatory status. Therefore, inconsistencies across different cohorts may reflect both genuine biological heterogeneity and differences in study subjects and methodological backgrounds. Third, there are limitations in extrapolating animal models to human pathology. Rodent models such as SPS provide important experimental foundations for exploring the gut microbiota–immune–brain axis (157), but they fail to fully recapitulate the intrusive memories, complex cognitive appraisals, social functional impairments, and long-term disease course of human PTSD (158). Consequently, animal models are more suitable as tools for generating and preliminarily validating mechanistic hypotheses rather than serving as direct proxies for human clinical evidence. Fourth, technical standards for microbiome research are not yet unified. The gut microbiome is influenced by multiple factors including age, diet, geographic environment, host genetics, and sample processing procedures (159). Furthermore, differences in sample collection, storage, DNA extraction, sequencing platforms, data quality control, and bioinformatics analysis across studies can compromise the comparability and reproducibility of results (160). Fifth, the specificity for PTSD still needs further clarification. Changes such as low alpha diversity, elevated IL-6, impaired intestinal barrier or BBB function, and microglial activation are not unique to PTSD but are also observed in depression, anxiety, chronic stress, and other inflammation-related diseases (64).

Therefore, future studies should include individuals with depression, anxiety, chronic stress, and trauma-exposed but non-PTSD populations as controls, and conduct stratified analyses based on symptom dimensions, trauma types, and illness stages to identify biological patterns specifically linked to PTSD persistence, abnormal fear memory, and impaired post-traumatic recovery. Overall, the primary bottleneck in current research is not whether associations exist, but the need to further elucidate the directionality, stability, disease relevance, and generalizability of these associations. Future improvements are needed in causal inference, cohort design, model selection, technical standards, disease-specific comparisons, and alignment of cited evidence with conclusion strength to advance research on the gut microbiota–immune–brain axis to a phase characterized by enhanced explanatory power and translational potential.

### 8.2 Future directions and translational pathways

Future research must first address the temporal relationship between gut microbiota changes, immune abnormalities, and the course of PTSD. Current cross-sectional studies make it difficult to determine whether microbiota dysbiosis and inflammatory activation occur before trauma exposure, in the early post-traumatic period, or after the establishment of chronic PTSD. Therefore, prospective longitudinal cohort studies are particularly crucial.

Ideally, studies should enroll individuals as early as possible after trauma exposure and serially collect fecal, blood, and clinical assessment data during the acute stress period, symptom evolution phase, and chronic stage, to map the dynamic relationship between microbiota characteristics, inflammatory markers, and symptom trajectories (32). Second, multi-omics integration is an important direction for moving this field from descriptive to mechanistic research. Metagenomics can identify microbial functional pathways related to metabolite synthesis, metabolomics can further validate changes in key molecules such as short-chain fatty acids and tryptophan metabolites, and immunomics helps delineate peripheral inflammatory profiles and immune cell states (161–163). Building on this foundation, the integration of neuroimaging, behavioral data, and symptom dimensions will facilitate the development of a comprehensive model linking microbial alterations to immune dysregulation and subsequent brain functional changes. Additionally, *in vitro* experimental platforms can serve as complementary tools for mechanism validation. Co-culture systems of brain organoids and gut organoids, microfluidic chips, and other models can model the interplay among microbial metabolites, intestinal barrier function, and neuroinflammation to some extent (164). However, such platforms are currently more suitable for mechanism refinement and hypothesis validation and should not be interpreted as pathways that can directly facilitate near-term clinical translation.

**Figure 6:**
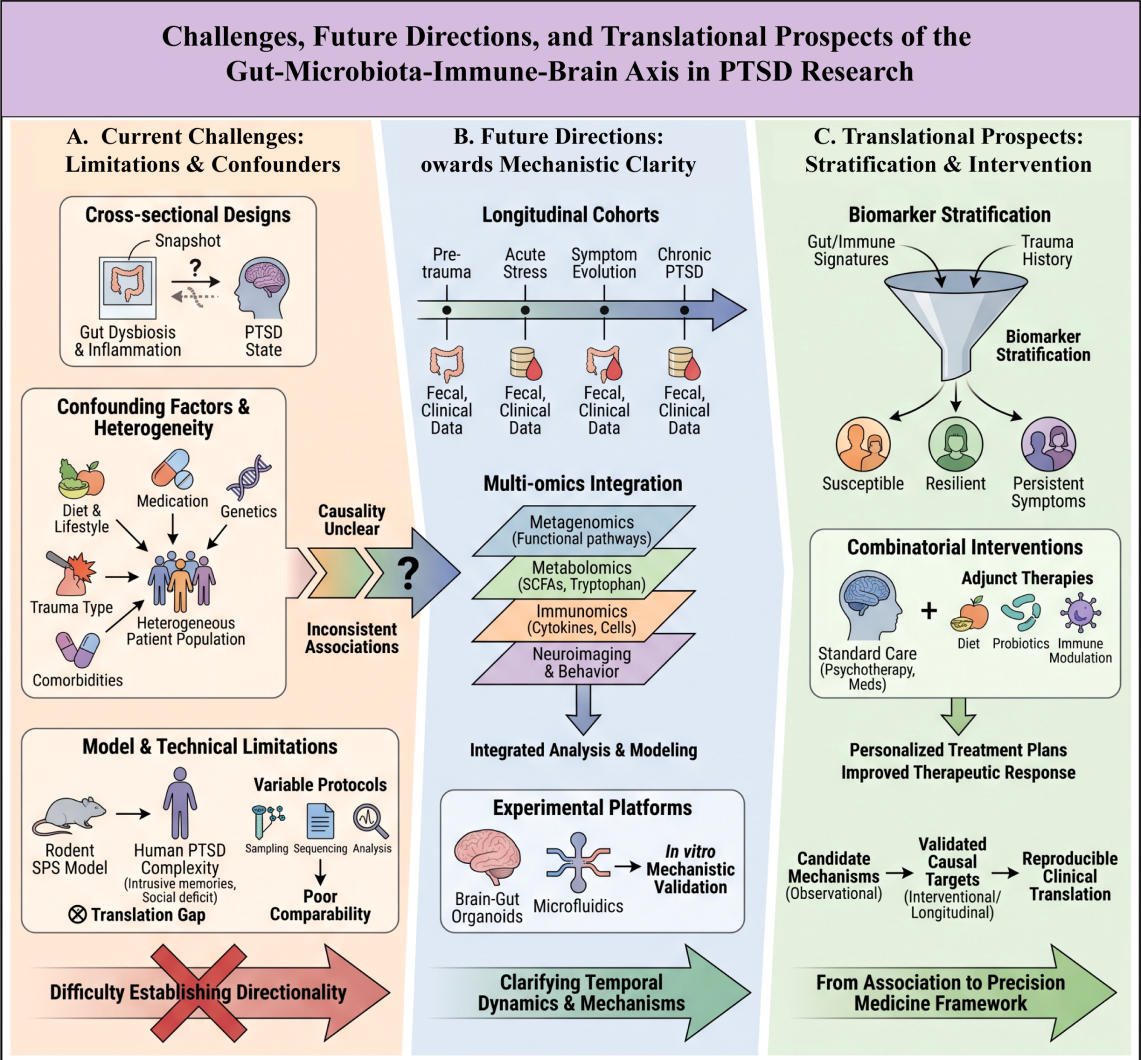
Evidence gaps, future directions, and translational pathways in gut microbiota–immune–brain axis research in PTSD. This figure outlines the main challenges in the field, including unclear causal direction, patient heterogeneity, limited extrapolation from animal models, non-uniform technical standards, and insufficient PTSD specificity. Future research should prioritize robust longitudinal cohorts, multi-omics integration, mechanism validation, and stratified intervention designs to advance this field from correlational studies to develop disease models with enhanced rigor and translational potential.

From a translational medicine perspective, the more realistic application prospects in this field mainly include three aspects. First, biological stratification based on microbiome and peripheral immune characteristics may help identify heterogeneous subgroups of PTSD, but its value lies in complementing clinical stratification rather than replacing existing psychiatric diagnoses. Second, combined intervention designs may better align with the complex pathological features of PTSD than single microbiome-based or anti-inflammatory strategies. For example, in patients with specific inflammatory phenotypes or more pronounced microbiota imbalances, the combined application of dietary adjustments, probiotics, or immunomodulatory strategies with standard psychotherapy and existing pharmacotherapy could be explored (165). Third, the key to future translational work is not to continue discovering more microbiota differences or inflammatory pathways, but to clarify, through longitudinal cohorts, interventional studies, and multi-omics integration, which changes have stable predictive significance, which mechanisms genuinely participate in symptom formation and maintenance, and which patient subgroups may benefit from microbiome-based or immunomodulatory interventions (**Figure 6**).

## 9 Conclusion

Pathophysiological research on PTSD is expanding from a central nervous system-dominated explanatory framework to an integrated perspective encompassing the interactions among the gut microbiome, immune system, and brain function. Current evidence suggests that PTSD is not only associated with abnormalities in fear memory circuits and dysregulation of stress responses but may also involve multi-level changes such as gut microbiota dysbiosis, peripheral low-grade inflammation, and neuroinflammation. Thus, the “gut microbiome–immune–brain axis” can be considered a candidate framework for reconceptualizing the multi-system abnormalities in PTSD, thereby offering a supplementary perspective for explaining some of the biological heterogeneity of PTSD through peripheral physiological imbalances. However, although existing evidence supports the significant value of this axis in explaining the multi-system pathological features of PTSD, its evidentiary boundaries should still be interpreted cautiously. Currently, related research is largely based on observational associations, preclinical models, and mechanistic inferences, characterized by a lack of direct human evidence, determination of causal direction, and reproducibility across different studies. In particular, the temporal relationship between changes in gut microbiota, immune abnormalities, and symptom manifestations has not been fully clarified, and the issue of inconsistent results across different cohorts indicates that this field is still significantly influenced by clinical heterogeneity, confounding factors, and differences in technical methods. Therefore, when interpreting this axis in the future, researchers must avoid conflating population correlations, animal mechanistic evidence, and translational application prospects, but instead evaluate their reliability and clinical implication separately based on study design and evidence hierarchy. At the same time, it should be recognized that gut microbiota dysbiosis, inflammatory activation, and neuroinflammation are not unique to PTSD. Future research needs to place PTSD within a comparative framework of depression, anxiety, and chronic stress-related disorders, focusing on identifying characteristic combinations related to trauma exposure, fear memory abnormalities, hyperarousal, and symptom persistence, rather than regarding single microbial taxa or inflammatory markers as specific biomarkers for PTSD. In the future, advancing this framework from a mechanistic hypothesis to a clinically verifiable model will depend on higher-quality longitudinal cohort studies, multi-omics integrative analyses, and stratified intervention designs. Compared to merely accumulating more correlational evidence, the more critical task is to identify stable and predictive microbial and immune features, clarify which mechanisms have stable and reproducible associations with the onset, maintenance, or persistence of PTSD symptoms, and further determine whether they have causal roles, while also exploring which patient subgroups are more likely to benefit from microbiome or immunomodulatory interventions. With the continuous improvement in causal inference capabilities and stratified study designs, the gut microbiome–immune–brain axis is expected to provide a more biologically grounded supplementary perspective for the mechanistic research and clinical management of PTSD.
